# New species and records of *Zora* C.L. Koch, 1847 (Araneae, Miturgidae) from Armenia

**DOI:** 10.3897/zookeys.1287.203444

**Published:** 2026-07-31

**Authors:** Alireza Zamani, Armine Kosyan, Yuri M. Marusik

**Affiliations:** 1 Zoological Museum, Department of Biodiversity Sciences, University of Turku, 20500 Turku, Finland Department of Zoology & Entomology, University of the Free State Bloemfontein South Africa; 2 Faculty of Biology, Yerevan State University, Yerevan, Armenia Faculty of Biology, Yerevan State University Yerevan Armenia; 3 Institute for Biological Problems of the North, FEB RAS, Portovaya Str. 18, Magadan 685000, Russia Institute for Biological Problems of the North, FEB RAS Magadan Russia; 4 Department of Zoology & Entomology, University of the Free State, Bloemfontein 9300, South Africa Zoological Museum, Department of Biodiversity Sciences, University of Turku Turku Finland

**Keywords:** Fauna, new records, spiders, taxonomy, Transcaucasia

## Abstract

New taxonomic and faunistic data on the miturgid spider genus *Zora* C.L. Koch, 1847 in Armenia are provided. Two species, *Z. armeniaca***sp. nov**. (♂♀) and *Z. bicoloripes***sp. nov**. (♂), are described as new to science and illustrated, and two species, *Z. pardalis* Simon, 1878 and *Z. spinimana* (Sundevall, 1833), are newly recorded from Armenia.

## Introduction

*Zora* C.L. Koch, 1847 is a genus of the spider family Miturgidae Simon, 1886 comprising 22 currently known species, distributed exclusively in the Holarctic: 20 species occur in the Palaearctic, whereas two are endemic to the Nearctic (WSC 2026). In Eurasia, the genus ranges northward to 70°N in Fennoscandia (Koponen et al. 2013) and to 62°N in eastern Siberia (Marusik et al. 1992). The fauna of the North Caucasus was recently revised by Ponomarev et al. (2024), who recognized 12 species in the region, including three that they described as new to science.

No revision has been published for the species occurring in the South Caucasus, and records from this area have mostly been reported as parts of general faunistic surveys. At present, three species are known from Azerbaijan (Nuruyeva and Zamani 2025), six from Georgia (Nentwig et al. 2026), and only one species, *Z. silvestris* Kulczyński, 1897, from Armenia (Mikhailov and Fet 1986).

Although more than half of the known species diversity of the genus has already been documented in the Caucasus, examination of material collected in Armenia revealed two additional species new to science, as well as two species recorded from the country for the first time. These findings are reported and described herein.

## Material and methods

Photographs of the Armenian specimens and their copulatory organs were taken using an Olympus Camedia E-520 mounted on an Olympus SZX16 stereomicroscope at the Zoological Museum of the University of Turku, Finland. Digital images captured at multiple focal planes were stacked using Helicon Focus 8.1.1. The endogyne was photographed after digestion of soft tissues in a 10% aqueous KOH solution. Comparative photographs of *Z. manicata* Simon, 1878 and *Z. silvestris*, taken by Pierre Oger, are available online at https://arachno.piwigo.com/. Leg measurements were taken dorsally and are presented as follows: total length (femur, patella, tibia, metatarsus, tarsus). All measurements are given in millimetres.

### Abbreviation

**RTA**—retrolateral tibial apophysis.

### Depositories

**CPO**—Collection of Pierre Oger, Héron, Belgium; **ZMMU**—Zoological Museum of Moscow University, Russia (K.G. Mikhailov); **ZMUT**—Zoological Museum of the University of Turku, Finland (V. Vahtera).

## Taxonomy

### Family Miturgidae Simon, 1886

#### Genus Zora

Taxon classificationAnimaliaAraneaeMiturgidae

C.L. Koch, 1847

7FA8BDB8-AAAF-52FF-B583-0312A590BEF9

##### Type species.

*Lycaena spinimana* Sundevall, 1833, from Sweden.

#### Zora armeniaca sp. nov.

Taxon classificationAnimaliaAraneaeMiturgidae

94B000E2-BB99-5CA9-B545-8CD384EE424C

https://zoobank.org/8260DFD2-3691-408F-B2EB-C17B5408457A

**Figure 1.**
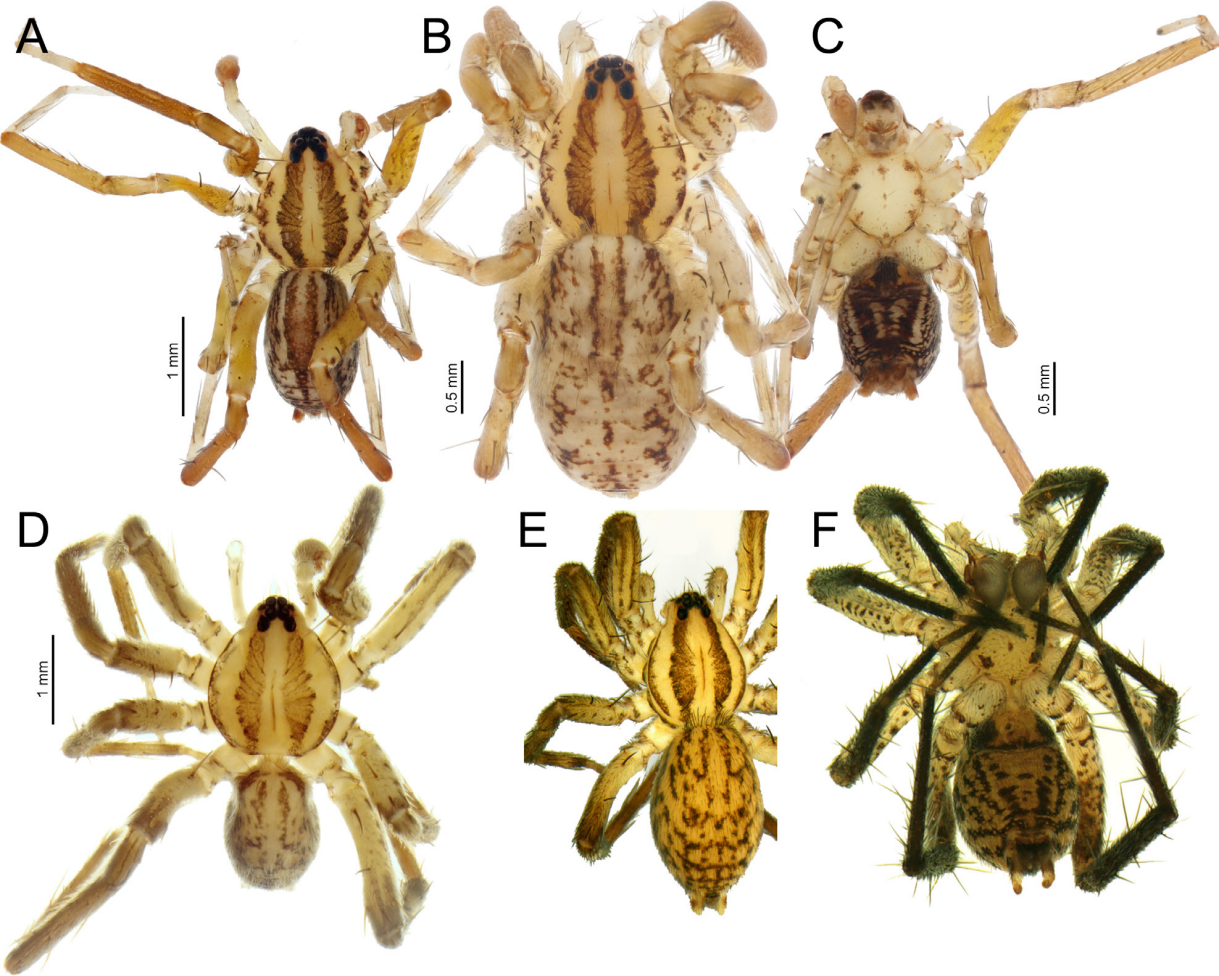
Habitus of *Zora armeniaca* sp. nov. (**A–C**) and *Z. silvestris* (**D–F**), in dorsal (**A, B, D, E**) and ventral (**C, F**) views. **A, C, D, F**. Males; **B, E**. Females. **D–F**. Courtesy of Pierre Oger.

**Figure 2.**
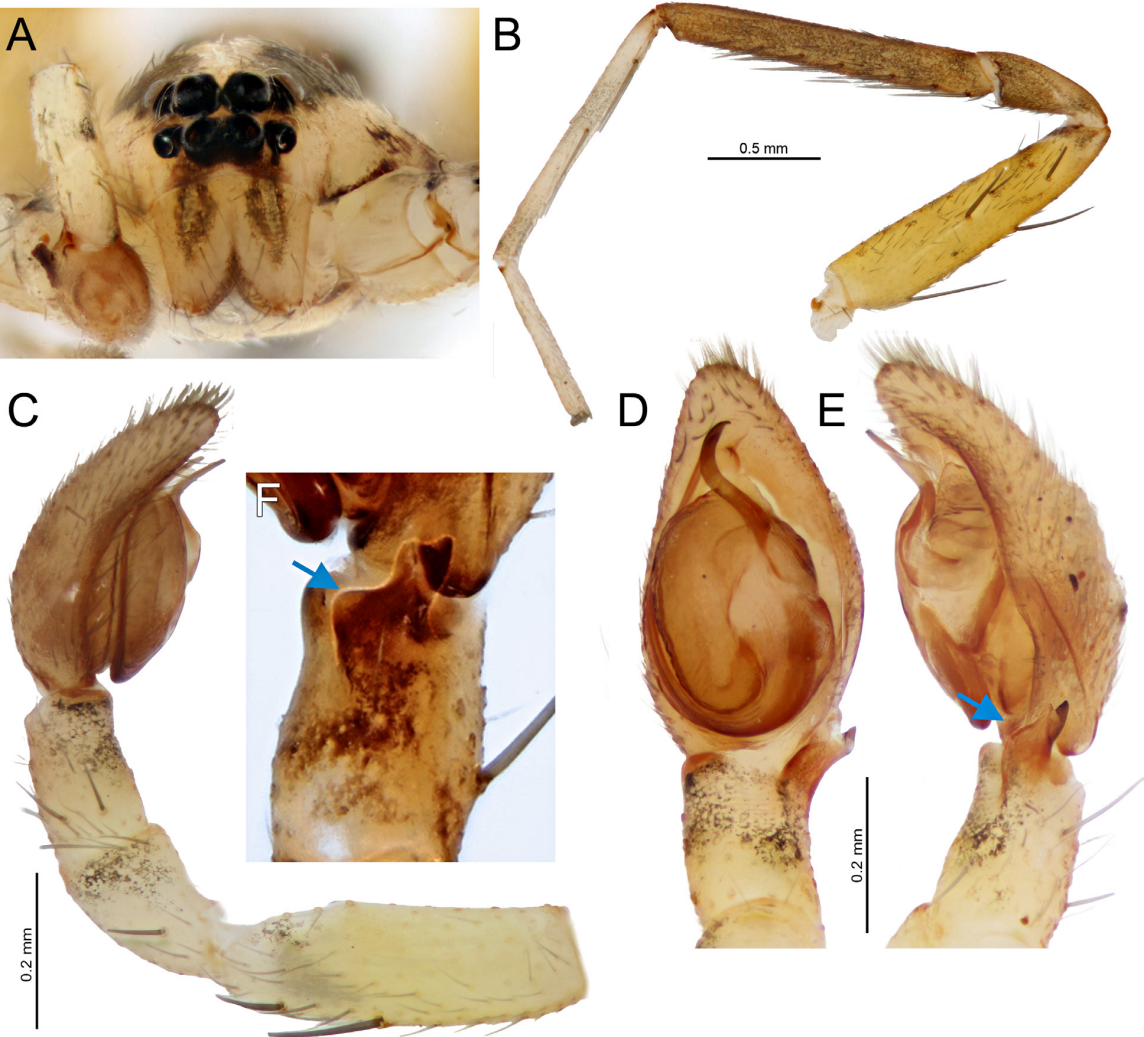
Males of *Zora armeniaca* sp. nov. (**A–E**) and *Z. silvestris* (**F**). **A**. Habitus, frontal view; **B**. Left leg I, retrolateral view; **C–E**. Palp, prolateral, ventral and retrolateral views; **F**. RTA, retrolateral view. Arrows indicate differences in the shape of the base of the RTA between the two species. **F**. Courtesy of Pierre Oger.

**Figure 3.**
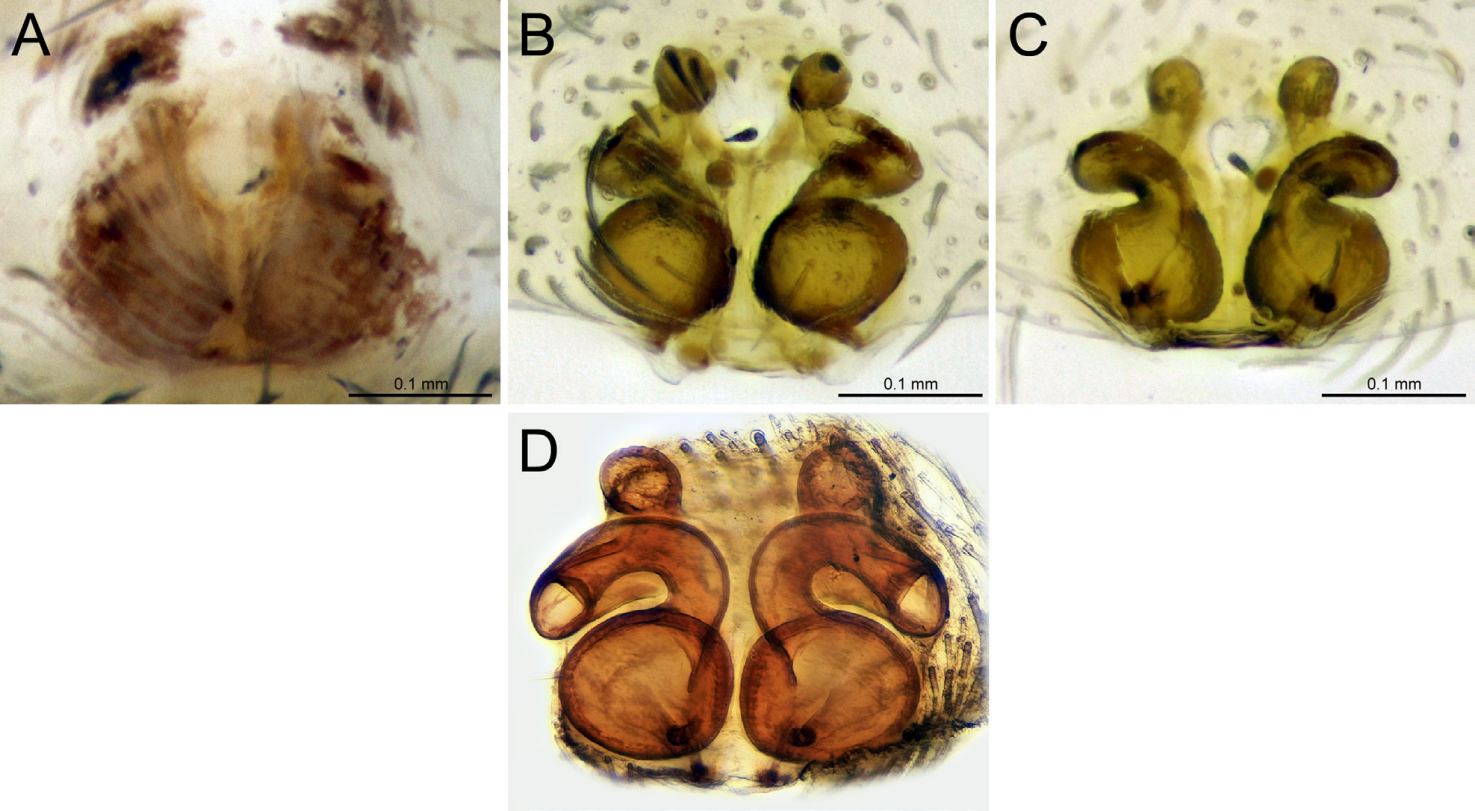
Epigynes of *Zora armeniaca* sp. nov. (**A–C**) and *Z. silvestris* (**D**). **A**. Intact, ventral view; **B**. Macerated, ventral view; **C, D**. Vulva, dorsal view. **D**. Courtesy of Pierre Oger.

Figs 1A–C, 2A–E, 3A–C

##### Type material.

***Holotype*** • ♂ (ZMMU), Armenia: Ararat Prov.: env. of Urtsadzor Vill., 39°54'N, 44°50'E, 1200–1300 m, clay slope, 6.v.2021 (Y.M. Marusik). ***Paratypes***: • 1♂ (ZMUT), env. of Goravan Vill., Goravan Sands State Sanctuary, 39°53'N, 44°43'E, 6.v.2021 (Y.M. Marusik); • 1♂ (ZMUT), Garni Gorge, Azat River, 40°06'N, 44°43'E, 1240 m, 5.v.2021 (Y.M. Marusik); Vayot Dzor Prov.: • 1♂2♀ (ZMMU), Gnishik R. canyon, rd. to Noravank Monastery, 39°41'N, 45°13'E, 1400 m, 10.v.2021 (Y.M. Marusik); Yerevan: • 1♂2♀ (ZMUT), botanical garden, 40°12'N, 44°33'E, 17.v.2021 (Y.M. Marusik).

##### Comparative material.

*Z. silvestris* (Figs 1D–F, 2F, 3D) — Belgium: Namur Prov.: • 1♀ (CPO), Sclaigneaux Nature Reserve, 50°30'N, 4°58'E, 2.v.2011; 1♂ (CPO), same locality, 19.v.2012; • 1♂ (CPO), Nismes, 23.v.1982 (R. Kekenbosch). France: Mayenne Dept.: • 1♀ (CPO), Thorigné-en-Charnie, Jardin de la Grange, 21.iv.–13.v.2013 (J.J. Tilly).

##### Etymology.

The specific epithet refers to the distribution of the new species in Armenia; adjective.

##### Diagnosis.

Among the species occurring in the Caucasus, the following possess two pairs of ventral spines on metatarsi I and II: *Z. alexeevi* Ponomarev, Mikhailov & Shmatko, 2024; *Z. alpina* Kulczyński, 1915; *Z. caucasia* Ponomarev, Mikhailov & Shmatko, 2024; *Z. dagestana* Ponomarev, Mikhailov & Shmatko, 2024; *Z. manicata*; *Z. osetica* Ponomarev, 2021; and *Z. silvestris*. Among these, the new species differs from *Z. caucasia*, *Z. manicata*, and *Z. osetica* in its lighter overall body coloration (cf. Fig. 1A–C vs. Ponomarev et al. 2024: figs 15, 16, 35, 43). In the shape of the RTA, it differs from *Z. alpina* and *Z. dagestana* (cf. Fig. 2E and Ponomarev et al. 2024: figs 19, 22), but resembles *Z. alexeevi* and *Z. silvestris*. It differs from *Z. silvestris* in the coloration of the carapace and legs, as well as in the morphology of the copulatory organs. In both sexes, the dark marginal stripe of the carapace is interrupted (vs. continuous), and the metatarsi and tarsi are not darkened (vs. darkened) (cf. Figs 1A–C, 2Bvs. Fig. 1D–F). The male differs from those of both *Z. alexeevi* and *Z. silvestris* in having the base of the RTA inclined (vs. forming a right angle; cf. Fig. 2E vs. Fig. 2F and Ponomarev et al. 2024: fig. 2). Among the light-colored species in the region, the female of *Z. dagestana* is unknown. Within the remaining species, the female of the new species differs from *Z. alpina* in the shape of the fovea (cf. Fig. 3B and Ponomarev et al. 2024: fig. 23), but resembles those of *Z. alexeevi* and *Z. silvestris*. It differs from both of the latter species in having shorter copulatory ducts (cf. Fig. 3C vs. Fig. 3D and Ponomarev et al. 2024: fig. 4).

##### Description.

**Male** (holotype). Habitus as in Figs 1A, 1C, 2A. Total length 2.85. Carapace 1.40 long, 1.15 wide. Carapace pale yellowish brown, with greyish brown submedian bands and lateral markings; chelicerae, maxillae, labium, and sternum pale yellowish brown; chelicerae with greyish brown longitudinal marking anteriorly (Fig. 2A); sternum with greyish brown median spots and lateral patches. Palps pale yellowish brown; legs light yellow with dark spots at femora, greyish brown to light brown at patellae and tibiae, and pale beige at metatarsi and tarsi. Abdomen dorsally with brown cardiac mark and posterior chevrons, otherwise with scattered pale grey and dark brown irregular markings; spinnerets brown, paler apically. Measurements of legs: I: 5.35 (1.30, 0.65, 1.40, 1.25, 0.75); II: 4.65 (1.15, 0.50, 1.25, 1.10, 0.65); III: 4.45 (1.15, 0.55, 1.05, 1.15, 0.55); IV: 6.65 (1.75, 0.60, 1.70, 1.85, 0.65). Paired ventral spines on metatarsi I, II: 2p.

Palp as in Fig. 2C–E; RTA curved in ventral view, with broad base having inclined distal margin, tip bifurcated: ventral branch digitiform in lateral view, dorsal branch sclerotized, folded; cymbial fold long, ca 0.4 as long as cymbium; base of tegular apophysis located at 3 o’clock position, directing prodistad and curving retrodistad at 12 o’clock position, tip tapering and slightly hooked; conductor hyaline, flat, partly covered by tegular apophysis; embolus base located at 3 o’clock position, broad, terminating at 12 o’clock position.

**Female** (paratype from Vayot Dzor). Habitus as in Fig. 1B. Total length 3.90. Carapace 1.70 long, 1.30 wide. Overall coloration and ventral spines on metatarsi I and II as in male; femora more distinctly mottled. Measurements of legs: I: 5.15 (1.50, 0.70, 1.40, 1.00, 0.55); II: 4.85 (1.40, 0.75, 1.25, 0.90, 0.55); III: 4.50 (1.25, 0.65, 1.05, 1.00, 0.55); IV: 6.75 (1.80, 0.75, 1.70, 1.80, 0.70).

Epigyne as in Fig. 3A–C; fovea forming heart-shaped figure anteriorly, narrowing medially with parallel margins, expanding posteriorly; copulatory ducts broad, making single medial loop before connecting to anterior margins of receptacles; receptacles circular, closely spaced.

##### Distribution.

Known only from the listed localities in Ararat, Vayot Dzor, and Yerevan, Armenia.

#### Zora bicoloripes sp. nov.

Taxon classificationAnimaliaAraneaeMiturgidae

0D63B393-FD57-509A-B075-678677403730

https://zoobank.org/6FFE77EC-C82A-402A-A515-311595C2EFEB

Figs 4A–D, 5A–C

##### Type material.

***Holotype*** • ♂ (ZMMU), Armenia: Ararat Prov.: Garni Gorge, Azat River, 40°06'N, 44°43'E, 1240 m, 5.v.2021 (Y.M. Marusik).

##### Comparative material.

**Figure 4.**
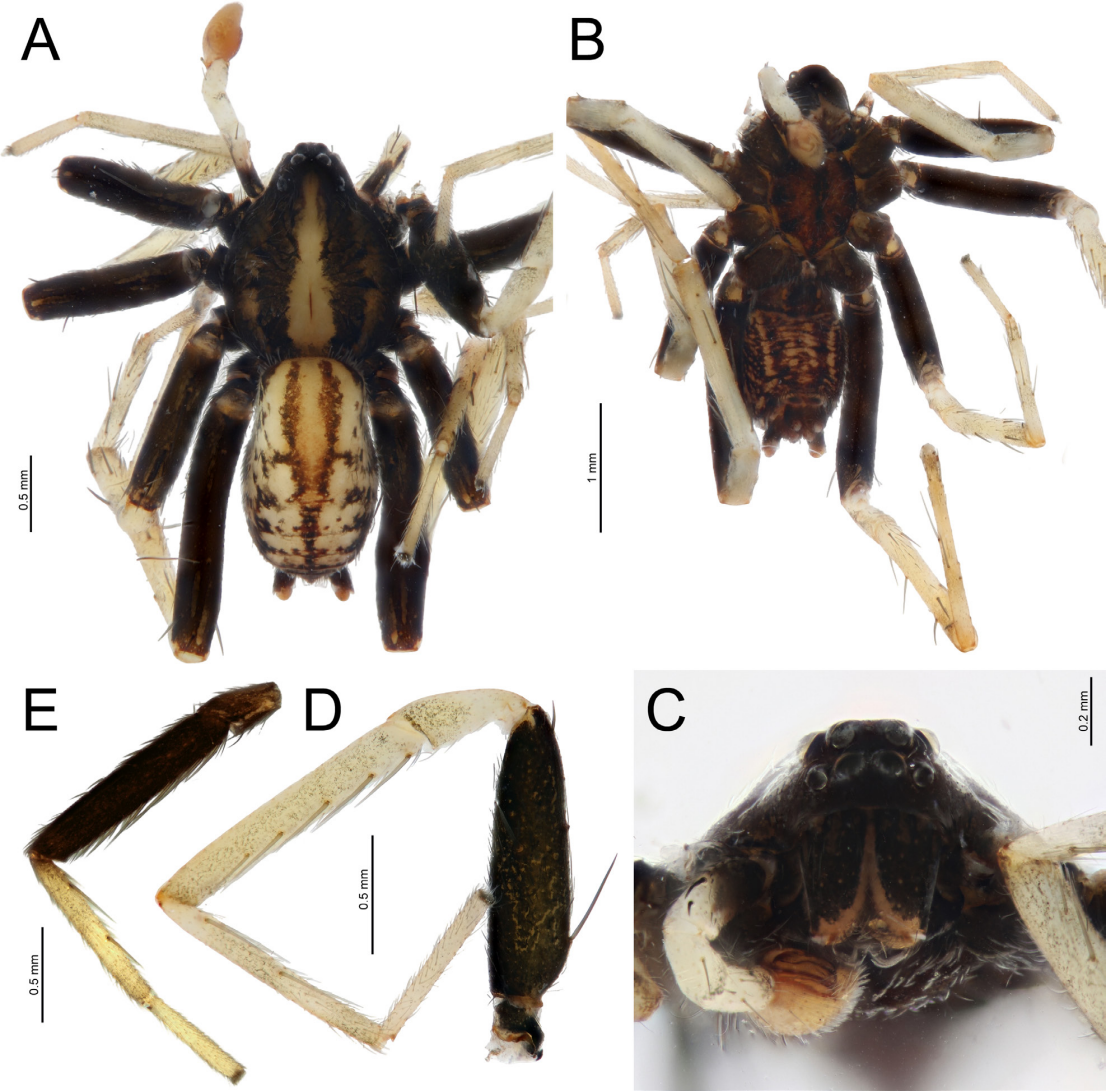
Males of *Zora bicoloripes* sp. nov. (**A–D**) and *Z. manicata* (**E**). **A, B, C**. Habitus, dorsal, ventral and frontal views; **D, E**. Left leg I, retrolateral view. **E**. Courtesy of Pierre Oger.

*Z. manicata* (Fig. 4E) — France: Savoie Dept.: • 1♂ (CPO), Saint-François-Longchamp, Le Perelles, 1720 m, scree with large boulders and heathland, 10.vii.2016 (A. Miquet).

##### Etymology.

The specific epithet is derived from Latin, meaning “two-colored legs,” and refers to the contrasting coloration of the legs in this species; adjective.

##### Diagnosis.

**Figure 5.**
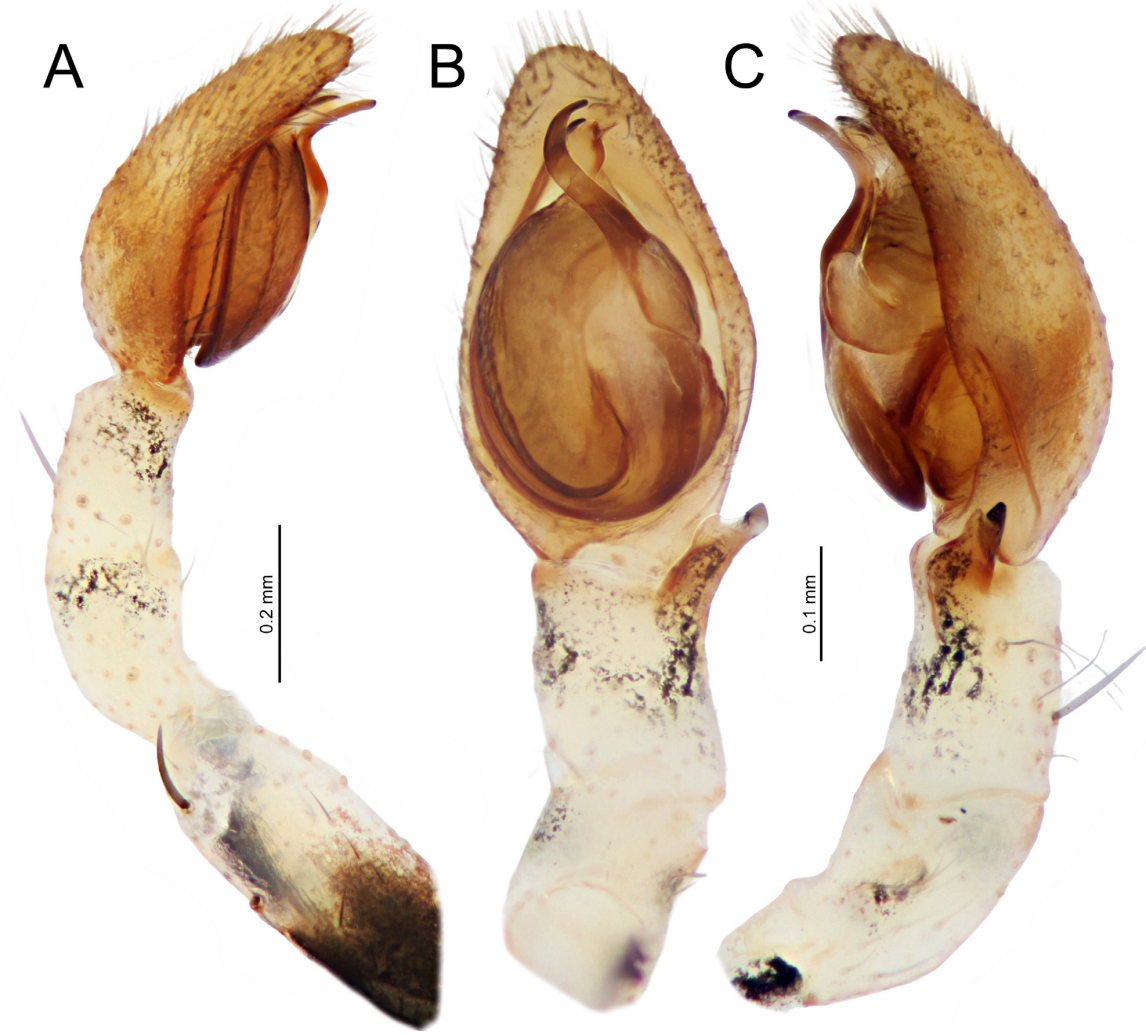
Male palp of *Zora bicoloripes* sp. nov. **A**. Prolateral view; **B**. Ventral view; **C**. Retrolateral view.

Among the species occurring in the Caucasus that have two pairs of ventral spines on metatarsi I and II, the new species resembles *Z. manicata* and *Z. osetica* in overall body coloration and the shape of the RTA. It differs from *Z. manicata* in having only the femora darkened, with the remaining leg segments light-colored (vs. only the metatarsi and tarsi light-colored; cf. Fig. 4C vs. Fig. 4E). *Zora bicoloripes* sp. nov. can be distinguished from *Z. osetica* by having five pairs of ventral tibial spines (vs. six), a subdivided tip of the RTA (vs. undivided; cf. Fig. 5C vs. Ponomarev et al. 2024: fig. 42), black femora on all legs (vs. brown), and a lighter abdomen with fewer brown spots (cf. Fig. 4A–C vs. Ponomarev et al. 2024: fig. 43).

##### Description.

**Male** (holotype). Habitus as in Fig. 4A, B, D. Total length 2.80. Carapace 1.45 long, 1.15 wide. Carapace pale yellowish brown, with dark brown broad submedian and lateral bands; chelicerae, maxillae, and labium dark brown; chelicerae with yellowish brown longitudinal marking promarginally (Fig. 4C); sternum reddish brown with black median band and lateral markings. Palps pale yellow; proximal 2/3 of femur dark brown and cymbium reddish brown. Legs light yellow; femora dark brown. Abdomen dark brown; dorsally with light brown cardiac mark bordered with brown stripe, beige submedian bands, and dark brown markings and posterior chevrons; ventrally with light brown lateral stripes and median spots; spinnerets dark brown, apically paler. Measurements of legs: I: 4.70 (1.15, 0.55, 1.25, 1.05, 0.70); II: 4.55 (1.20, 0.55, 1.05, 1.05, 0.70); III: 4.20 (1.15, 0.50, 1.00, 1.00, 0.55); IV: 6.10 (1.60, 0.55, 1.60, 1.65, 0.70). Paired ventral spines on metatarsi I, II: 2p.

Palp as in Fig. 5A–C; as described for the previous species.

**Female**. Unknown.

##### Distribution.

Known only from the type locality in Ararat, Armenia.

#### Zora pardalis

Taxon classificationAnimaliaAraneaeMiturgidae

Simon, 1878

67408316-1A5E-5251-9D98-1DD55BBDB3B4

Zora pardalis : Ponomarev et al. 2024: 601, figs 48–51 (♂♀). For a list of eight taxonomic entries, see WSC (2026).

##### Material.

Armenia: Kotayk Prov.: • 1♂ (ZMMU), env. of Aghavnadzor, 40°33'N, 44°40'E, 14.v.2021 (Y.M. Marusik).

##### Distribution.

West Palaearctic: from Iberian Peninsula to Kazakhstan. New record for Armenia.

#### Zora spinimana

Taxon classificationAnimaliaAraneaeMiturgidae

(Sundevall, 1833)

26743638-8B24-5D5D-9BBA-C406B2658AA9

Zora spinimana : Ponomarev et al. 2024: 603, figs 56–60 (♂♀). For a list of 50 taxonomic entries, see WSC (2026).

##### Material.

Armenia: Kotayk Prov.: • 2♂6♀3juv. (ZMMU), env. of Aghavnadzor, 40°33'N, 44°40'E, 14.v.2021 (Y.M. Marusik).

##### Distribution.

Trans-Palaearctic: from Iberian Peninsula to Kamchatka. New record for Armenia.

## Discussion

Based on the results of the present study, five species of *Zora* are now known from Armenia, including two described here as new to science and two newly recorded for the country; previously, only *Z. silvestris* had been reported from Armenia, based on a single, obscure record from Meghri (Mikhailov and Fet 1986). The recent revision of the Caucasian species by Ponomarev et al. (2024) did not include Armenia within the known range of *Z. silvestris*. It is possible that this record actually pertains to *Z. armeniaca* sp. nov., which closely resembles *Z. silvestris* in both the shape of the RTA and the coloration pattern.

There is remarkable homogeneity in the structure of the male palp and the epigyne among species of this genus, and species delimitation relies largely on somatic characters, including spination and color patterns. This raises the possibility that additional undescribed species have been overlooked or misidentified where somatic characters were not sufficiently considered during identification. Ponomarev et al. (2024) demonstrated that the diversity of the genus in the Caucasus has been underestimated and that revision of existing records and museum material from neighbouring countries would likely reveal additional undescribed species.

The two species described here represent the first members of the genus described from the South Caucasus. Apart from a record of *Z. osetica* from Georgia by Ponomarev et al. (2024), all other regional records belong to widely distributed species and may have been misidentified. Indeed, several species treated by Ponomarev et al. (2024) had previously been misidentified (e.g., *Z. caucasia* as *Z. silvestris* in North Ossetia and Adygeya, and as *Z. manicata* in Karachay-Cherkessia). Similarly, six species of *Zora* are currently known from Iran (Zamani 2025), which, apart from the endemic *Z. huseynovi* Zamani & Marusik, 2017 (known only from its type locality in northern Iran; Zamani and Marusik 2017), all have wide distributions and are likewise in need of revision.

## Supplementary Material

XML Treatment for Zora

XML Treatment for Zora armeniaca

XML Treatment for Zora bicoloripes

XML Treatment for Zora pardalis

XML Treatment for Zora spinimana

## Additional information

### Artificial Intelligence (AI) use

The authors accept full responsibility for the content of the manuscript, including the disclosure of any use of AI.

No AI tools were used in the preparation of this manuscript.

### Author ORCIDs

A. Zamani https://orcid.org/0000-0002-8084-9666

A. Kosyan https://orcid.org/0000-0001-5692-5369

Y.M. Marusik https://orcid.org/0000-0002-4499-5148

### Data availability

No datasets were generated or analyzed during the current study.
